# ID 169 - Viabilidade de Implantação do Monitoramento Terapêutico da Vancomicina em Hospitais de um Estado Brasileiro

**DOI:** 10.5327/2237-9622.2025.v34s1.169

**Published:** 2025-11-25

**Authors:** Kelli Carneiro de Freitas Nakata, Ternize Mariana Guenkka, Gilson Yugi Nakata, Jessica Weis Bonfanti, Leticia Rossetto da Silva Cavalcante, Maria do Carmo Souza

**Keywords:** monitoramento de medicamentos, vancomicina, análise de impacto orçamentário

## Abstract

**Introdução:**

A vancomicina é tratamento de escolha para resistente à meticilina. Entretanto, seu estreito índice terapêutico tem exigido monitoramento com o intuito de garantir eficácia clínica e minimizar nefrotoxicidade. O objetivo desse trabalho foi avaliar a viabilidade de implantação do monitoramento terapêutico da vancomicina nos hospitais estaduais de um estado brasileiro.

**Método:**

Uma síntese de evidências buscou responder à pergunta: o monitoramento terapêutico da vancomicina é seguro e eficaz quando medidos em termos de nefrotoxicidade e sucesso terapêutico em comparação ao não monitoramento? A busca por títulos foi realizada nas bases de dados PubMed; Cochrane Library e LILACS, usando os descritores: . Foram considerados estudos de revisão sistemática, sem restrição de língua. O software Rayyan foi utilizado para remoção de duplicatas e triagem, realizada por três revisores independentes, com base na leitura de títulos e resumos. A extração de dados deu-se em instrumento padronizado por dois pesquisadores. A qualidade metodológica dos estudos incluídos foi analisada pela ferramenta AMSTAR-2. Um impacto orçamentário, sob a perspectiva do pagador, foi calculado como a diferença entre os custos médios anuais do cenário hipotético de incorporação da tecnologia e o cenário atual, no qual não contempla o monitoramento terapêutico da vancomicina, levando-se em consideração a difusão da tecnologia ano a ano com base na adesão descrita na literatura. A população de interesse foi definida pelo método da demanda aferida com base no número médio de usuários de vancomicina nos anos de 2020, 2021 e 2022 nos hospitais estaduais. Os custos foram calculados com fulcro no curso clínico dos indivíduos, considerando apenas os custos diretos médicos relacionados à nefrotoxicidade. O modelo não considerou os benefícios da tecnologia em termos de eficácia clínica, tampouco a necessidade de terapia renal substitutiva no período pós-alta hospitalar. Um formulário estruturado foi enviado a cada hospital com o propósito de avaliar a capacidade instalada para uma possível incorporação da tecnologia.

**Resultados:**

O monitoramento terapêutico da vancomicina está associado a chances significativamente mais altas de eficácia clínica (OR = 2,62, IC 95% 1,345,11 p=0,516; I2=0%) e chances mais baixas de nefrotoxicidade (OR = 0,27, IC 95% 0,12–0,58 P = 0,461; I2=0%) quando comparados ao não monitoramento. O impacto orçamentário se mostrou negativo, indicando economia de recursos decorrentes da adoção da tecnologia avaliada. A economia pode variar de -R$ 23.945,83 no primeiro ano e de -R$ 42.144,65 no quinto ano. Em cinco anos, o impacto cumulativo pode ser de até -R$ 166.662,95. O estudo junto aos hospitais demonstrou que a implementação da tecnologia depende de treinamento de pessoal; fluxo de trabalho entre equipes; desenvolvimento de protocolos; uso de calculadoras específicas e dosagem sérica de vancomicina.

**Conclusão:**

O monitoramento terapêutico da vancomicina reduz as chances de lesão renal aguda induzida por vancomicina e, portanto, é recomendado por diversas diretrizes clínicas. Semelhante a outros hospitais brasileiros, a implantação dessa tecnologia é viável nos hospitais estaduais podendo trazer benefícios clínicos e econômicos, além de cooperar com ações ligadas à gestão de risco e segurança do paciente.

